# A New Calibration Methodology for Thorax and Upper Limbs Motion Capture in Children Using Magneto and Inertial Sensors

**DOI:** 10.3390/s140101057

**Published:** 2014-01-09

**Authors:** Luca Ricci, Domenico Formica, Laura Sparaci, Francesca Romana Lasorsa, Fabrizio Taffoni, Eleonora Tamilia, Eugenio Guglielmelli

**Affiliations:** 1 Laboratory of Biomedical Robotics and Biomicrosystems, Universitá Campus Bio-Medico di Roma, Via Àlvaro del Portillo 21, Rome 00128, Italy; E-Mails: d.formica@unicampus.it (D.F.); f.taffoni@unicampus.it (F.T.); e.tamilia@unicampus.it (E.T.); e.guglielmelli@unicampus.it (E.G.); 2 Institute of Cognitive Sciences and Technologies (ISTC), National Research Council (CNR), Via Nomentana 56, Rome 00161, Italy; E-Mails: laura.sparaci@istc.cnr.it (L.S.); francesca.lasorsa@yahoo.it (F.R.L.)

**Keywords:** magneto and inertial measurement unit, anatomical coordinate system, functional frame definition, calibration protocol, children motion capturing

## Abstract

Recent advances in wearable sensor technologies for motion capture have produced devices, mainly based on magneto and inertial measurement units (M-IMU), that are now suitable for out-of-the-lab use with children. In fact, the reduced size, weight and the wireless connectivity meet the requirement of minimum obtrusivity and give scientists the possibility to analyze children's motion in daily life contexts. Typical use of magneto and inertial measurement units (M-IMU) motion capture systems is based on attaching a sensing unit to each body segment of interest. The correct use of this setup requires a specific calibration methodology that allows mapping measurements from the sensors' frames of reference into useful kinematic information in the human limbs' frames of reference. The present work addresses this specific issue, presenting a calibration protocol to capture the kinematics of the upper limbs and thorax in typically developing (TD) children. The proposed method allows the construction, on each body segment, of a meaningful system of coordinates that are representative of real physiological motions and that are referred to as functional frames (FFs). We will also present a novel cost function for the Levenberg–Marquardt algorithm, to retrieve the rotation matrices between each sensor frame (SF) and the corresponding FF. Reported results on a group of 40 children suggest that the method is repeatable and reliable, opening the way to the extensive use of this technology for out-of-the-lab motion capture in children.

## 1. Introduction

The possibility of capturing and quantitatively measuring children's motion repertoire in a daily life scenario is of great interest for a number of reasons. Clinical evaluation tools to measuring motor skills in children are, to date, able to offer qualitative, rather than quantitative, evaluation (*i.e.*, studies using standardized measures have difficulties in providing fine-grained details on children movement properties). Examples of widely used test to measure motor skills in children are the Gross Motor Function Measure, the Movement ABC or the 10 Meter Walk Test [1–3]). Therefore, the lack of reliable, objective measurements foster interest in the development of tools to accurately capture information about children's motion skills in real-life environments. For instance, it would be of key importance in the rehabilitation of children with a chronic health condition, such as cerebral palsy, to guide and evaluate interventions, to monitor progress and also to provide families with objective feedback [4]. Besides, quantitative motion evaluation can support standard clinical rating scales, providing clinicians with enriched information on patients' health [5].

Furthermore, research studies on the role of motor and communicative gesture (e.g., gestures) have highlighted the importance of assessing the motor characteristics of children's behavior [6,7]. Furthermore, in children with autistic spectrum disorder (ASD) or “at high-risk” for ASD, appropriate motion evaluation tools may be of relevance for early diagnosis and intervention [8–10].

A considerable number of systems for human motion capturing is commercially available at present. Though the technologies and the approaches are many, exhibiting different performance characteristics and operating on entirely different physical principles, no ‘silver bullet’ currently exists [11]. Among the plethora of choices, wearable technologies have the potential to meet the requirements for this specific application, as reported in [12,13]. Wearable motion tracking systems are based on M-IMUs, which identify a class of devices comprising tri-axial accelerometers, gyroscopes and magnetometers. Besides the information provided by the single sensor (*i.e.*, acceleration, angular velocity and magnetic flux density), M-IMUs can provide and maintain an accurate 3D-orientation estimate thanks to sensor fusion algorithms (for a comprehensive review on this topic, see [14]).

In order to obtain a precise tracking of the kinematics of human joints, the fulfillment of a calibration protocol is strictly required. The aim of our research was to define such a calibration procedure to capture the kinematics of upper limbs and thorax in children. Our method permits the construction of meaningful functional frames (FFs), in the sense of being representative of real physiological motions, on each body segment and allow for estimating of the rotation matrices between each sensor frame (SF) and the corresponding FF. A typical calibration protocol is composed of the following steps: (1) a series of fixed reference postures and/or functional movements that the subject under experimentation is asked to perform; (2) the definition of both an FF on each body segment of interest and a mapping between each axis of the FF and each reference posture/functional movement; and (3) the computation of the transformation matrix between each FF and its corresponding SF. Despite existing literature proposing procedures for the kinematic tracking of both upper and lower limbs [15–19], no study to date has provided a calibration protocol specifically designed to be used with children. In fact, existing procedures do not take into consideration the constraints related to an use of M-IMU technology with children, e.g., the fact that particular care in the choice of movements to perform is required. Therefore an ad-hoc design is required. Based on the outcomes from a previous study [20], we have built a calibration protocol, which defines an ameliorated set of reference postures/functional movements, a new way to estimate reference axes from sensor data, and introduces a novel methodology to compute the transformation matrix. The experimental procedure has been tested in typical development (TD) children, and it has been administered by non-technicians in daily life scenarios (e.g., at school or at home), as it does not need any special expertise.

This paper is organized as follows: Section 2 provides an introduction of the motion tracking system architecture, including the hardware and software components that have been employed, and offers a detailed description of the proposed calibration protocol alongside data analysis methodology; Section 3 reports the results of the experimental session; Section 4 discusses the results and presents some conclusions.

## 2. Materials and Methods

### 2.1. System Architecture

As hardware, the experimental setup is comprised of a set of 5 wireless sensing units (SUs) chosen among the number of commercially available systems. In particular, we chose to use Opal by APDM Inc. (Portland, OR, USA), because their smaller dimension and lower weight (22 g) makes them particularly suitable for the target application. Each SU contains an M-IMU, a micro-SD for robust data logging and a radio transceiver. The orientation information is computed via the manufacturer's Kalman filter, in the form of a quaternion ( $q_{SF}^{G}$) relating the orientation of a global, Earth-based frame (G) to the SF. An access point is provided to gather synchronized sensor data and to make them available to a PC in real time.

**Figure 1.**
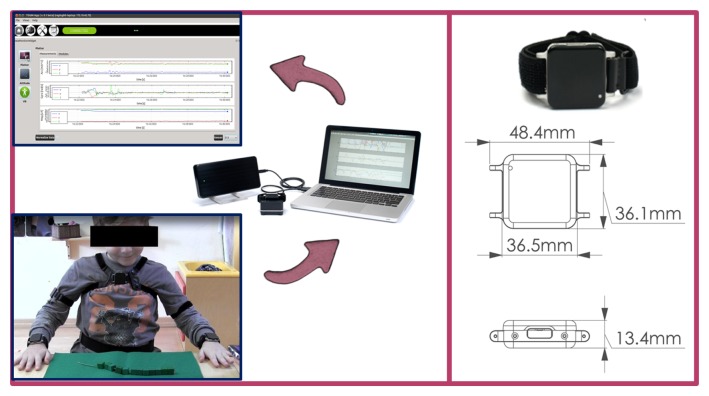
Experimental setup: the 5 sensing units (SUs) are attached to the body at predefined spots and data are collected and visualized via the developed software interface.

As software, we developed a C++ GUI application for agile system managing and data collection, using the Qtcross-platform framework. Each M-IMU sensor can be tagged within the software application with the name of the human joint to which it is attached in order to store this information in the data logs. A complete scheme of the experimental setup is shown in Figure 1.

### 2.2. Calibration Protocol

This section describes the calibration protocol for the kinematic tracking of thorax and upper limb motion in children. However, before providing details and in order to clarify what will follow, we shall provide an overview of the entire procedure.

The proposed methodology was tested on a group of 40 primary school children (the average age is 6.9 ± 0.65 years old; the minimum is 6.0 and the maximum is 8.0; and the group is composed of 22 females and 18 males). Informed consent was obtained from all the children' parents, as required by the Institutional Review Board at the National Research Council (CNR). An experimentation session took place in the school, thus capturing motion in an environment familiar to the children.

Before starting the experimentation session, being aware of the accelerometer and magnetometer calibration issues reported in [21], the calibration status of each sensor was assessed following the procedure described in [22]. Then, each sensor was fixed to the corresponding body segment of interest using Velcro straps. During the procedure, the mapping sensor-body segment was recorded in the data logs through the developed software interface.

**Figure 2.**
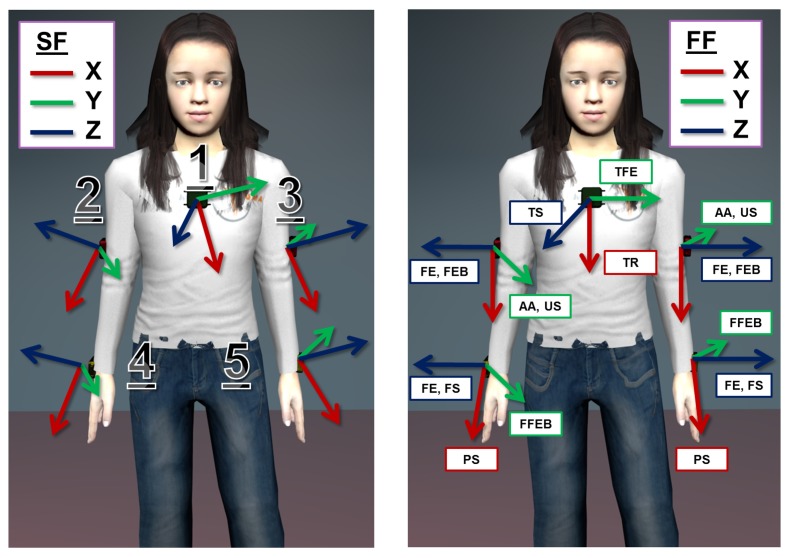
The figure on the left reports the position of the SUs on the body segments (1, thorax; 2, right upper arm; 3, left upper arm; 4, right forearm; 5, left forearm). On the right, a possible assignment of the functional frame (FF) on the body is reported. Note that each movement in the calibration protocol list is matched to an axis in the FFs (refer to Section 2.2.1. for the meaning of the acronyms). SF, sensor frame.

As a preliminary step, the calibration protocol requires 5 SUs to be attached to the following body spots: central on the thorax, latero-distally on the right and left upper arm and near the wrist on the right and left forearm, as shown in Figure 2. Furthermore, each body spot is assigned an arbitrarily fixed FF, which is descriptive of the kinematic of the body spot itself, e.g., the axes of the FF on the upper arm will be related to the degree of freedom of the shoulder joint. Finally, each SU is associated with a corresponding FF. Then, the actual calibration procedure articulates in a series of 4 successive steps:

- step 1: the participant, while wearing the SUs, completes a predefined list of movements and adopts a set of stances, separately comprising the thorax, the upper limbs or the forearms. Each movement in the list is associated with an axis of the involved FFs on the body.
- step 2: the information is collected from the SUs and pre-processed (*i.e.*, normalization) in order to extract the direction of the gravity vector and of the angular velocity vector, respectively, during the stationary postures and movements.
- step 3: an estimate of each axis of the FFs, relative to the corresponding SF, is obtained from the pre-processed data, applying singular value decomposition (SVD). Moreover, associated with the estimates is a measure of the reliability of the computed axis.
- step 4: given the set of FF axes and their estimates in the SFs, a regression algorithm, namely Levenberg–Marquardt (LM), is applied to compute the transformation between each pair of systems of coordinates. Furthermore, the reliability indices computed at step 3 are used to properly condition the regression algorithm.

The final aim of the calibration procedure is then to define the transformation between each SF and the corresponding, arbitrarily fixed FF, *i.e.*, the rotation matrix, $R_{SF}^{FF}$. Eventually, its estimation allows for the transition from the orientation information of the SUs to the kinematic description of the upper body.

#### 2.2.1. Calibration Movements

**Figure 3.**
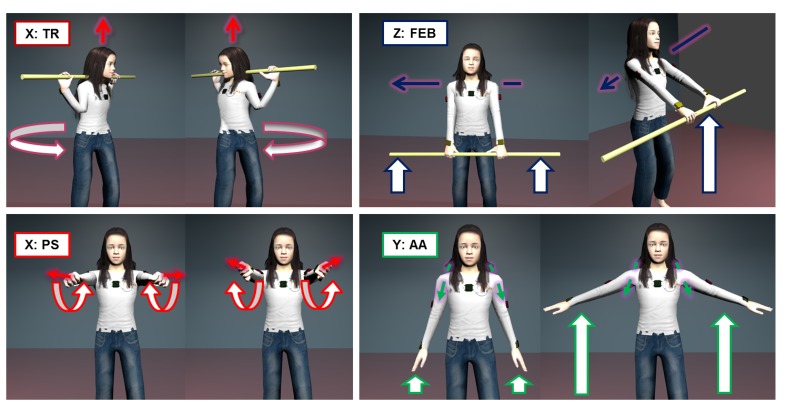
A subset of the calibration movements to be performed in the protocol. Clockwise from the top left we have: TR associated with the x-axis of *FF_Thorax_*; FEB of the upper arm associated with the z-axis of *FF_Upperarm_*; AA of the upper arms associated with the y-axis of *FF_Upperarm_*; PS of the forearms associated with the x-axis of *FF_Forearm_*.

The first part of the calibration itself consists of a set of stationary postures and mono-axial, functional movements that the participant has to perform. This approach relies on the two procedures that are commonly referred to in the literature as the “reference” and “functional” method, respectively [19]. The aim of this first step of the protocol is to allow for the identification of, at least, a pair of non-aligned axes on each FF of the body segments of interest. These axes are representative of certain directions of interest on the body, *i.e.*, the transverse axis of a body segment, or of physiological motion, *i.e.*, the axis of rotation of the shoulder joint during flexion-extension of the upper arm. For the kinematic tracking of the thorax and upper limbs in children, we propose the following calibration movement:

- **Thorax**
**TS**:The gravity vector measured in supine position with arms alongside the body and palms facing down (5 s)**TR**:Rotation of the thorax on the transverse plane while holding a bar (3–4 reps.), the movement is shown in Figure 3**TFE**:Flexion-extension from standing position with legs opened at shoulder-width (3–4 reps.)
- **Upper arm**
**US**:The gravity vector measured in supine position with arms alongside the body and palms facing down ((5 s)**AA**:Ab- and ad-duction from standing position with legs opened at shoulder-width (3–4 reps.); see Figure 3**FE**:Flexion-extension from standing position with legs opened at shoulder-width (3–4 reps.)**FEB**:Flexion-extension while holding a bar with hands at shoulder breadth with an adducted thumb grasp, as shown in Figure 3 (3–4 reps.)
- **Forearm**
**FS**:The gravity vector measured in supine position with arms alongside the body and palms facing down (5 s)**PS**:Pronation and supination movement with arms fully extended and hands closed (3–4 reps.) see Figure 3**FFEB**:Flexion-extension while holding a bar with hands at shoulder breadth and with upper arms close to the body (3–4 reps.)

All calibration movements were proposed to children as a short gym exercise. An adult played the role of coach, and children were asked to observe one movement sequence before proceeding to execute the movement together with the coach. The reported list of movements and stationary postures identifies a set of no less than 3 non-aligned axes for each body segment FF. The above list describes a single trial of the calibration protocol, and the complete version will be composed of a set of 3 trials.

#### 2.2.2. Data Collection

Aiming at identifying meaningful axes for each body segment, we are interested in collecting two kinds of information during the protocol trials, *i.e.*, accelerometer readings for the posture part and gyroscope readings for the dynamic part. In fact, accelerometers record the direction of the gravity vector while the subject is lying in supine position with palms facing down. The gyroscopes, instead, capture the angular velocity vector during movements, which allows one to identify the direction of the axis of rotation itself. Each single measurement from accelerometers or gyroscopes is a vector of data in ℝ^3^ made of the three axis sensor readings. At this stage, all collected data are normalized, *i.e.*, each measurement is transformed into a unit norm vector.

#### 2.2.3. Reference Axis Identification

We then proceeded to build the following measurements matrices, $A^{SF}={\left[a_{1}^{SF},a_{2}^{SF},\ldots ,a_{N}^{SF}\right]}^{T}\in {\mathbb{R}}^{N\times 3}$ and $\Omega^{SF}={\left[\omega_{1}^{SF},\omega_{2}^{SF},\ldots ,\omega_{N}^{SF}\right]}^{T}\in {\mathbb{R}}^{N\times 3}$, made of the **N** normalized readings from the accelerometers and the gyroscope, respectively. After that, we applied singular value decomposition (SVD):

(1) $$\begin{array}{c} \begin{array}{cc} \begin{array}{c} A^{SF}=U\Sigma (\sigma_{i})V^{T}\\ \Omega^{SF}=U\Sigma (\sigma_{i})V^{T} \end{array} & i=1,2,3 \end{array}\\ \begin{array}{ccc} U\in {\mathbb{R}}^{N\times N}, & \sum \in {\mathbb{R}}^{N\times 3}, & V\in {\mathbb{R}}^{3\times 3} \end{array} \end{array}$$

where **U** and **V** are the orthogonal matrices coming out from the decomposition and containing an orthogonal basis for ℝ*^N^* and ℝ^3^ spaces, respectively. Σ is a diagonal matrix with the singular values on the main diagonal (*σ_i_*). Based on the hypothesis of a stationary posture during the static part and a uni-axial movement during the dynamic part of the calibration protocol, the desired axis of reference will correspond to the right singular vector associated with the highest singular value (*σ*_1_ > *σ*_2_ > *σ*_3_), *i.e.*, the first column of **V**.

This result can be explained by adding the following considerations. First, in the ideal case of flawless, mono-axial movement, the angular velocity vectors will lay on a line in 3D Euclidean space, *i.e.*, they are contained in a subspace of dimension 1. Secondly, during the static part of the protocol, the projections of the gravity vectors on each axis of the SF are assumed to be constant. Again, this implies the accelerometers' readings to be confined in a subspace of dimension 1, specifically on a point. Therefore, both **A***^SF^* and **Ω** *^SF^* are expected to be rank 1 matrices. In practice, given the objective inability for a human being to perform a perfect mono-axial joint rotation, physiological movements while lying supine (e.g., movements due to breathing) and the sensor noise, **A***^SF^* and **Ω** *^SF^*, will be full-rank. Therefore, what can be achieved with SVD is a robust discrimination between the useful information and disturbances, to identify the underlying 1-rank submatrix and its basis, *i.e.*, the axis of rotation.

In addition, we used singular values to define an index of the reliability of the computed axis, given by the following expression:

(2) $$\begin{array}{ll} \rho =\frac{\sigma_{1}}{\sum_{i=1}^{3}\sigma_{i}}, & \left[\frac{1}{3}\leq \rho \leq 1\right] \end{array}$$

which is a dimensionless quantity representing the ratio of the largest singular value (*σ*_1_) and the sum of all the diagonal entries of the Σ matrix. This index provides an indication about the quality of the collected dataset, in terms of how data distributes along directions orthogonal to the computed axis of reference. In the ideal case, *ρ* should be the unity. In the practical one, the higher the value of *ρ*, the better will be the dataset collected.

By applying this procedure to all the datasets captured during steps 1 and 2, the outcome will be a set of pairs composed of axis estimates and the corresponding reliability index (*υ⃗_SF_*, *ρ*), for any movement in the calibration list. Further, for each FF defined on the body segments of interest, a set of at least two non-aligned axis estimates is available.

#### 2.2.4. Transformation Matrix Computation

With the purpose of identifying the 3D rotation matrix ( $R_{SF}^{FF}$) relating each SF to its corresponding FF, the axes estimates together with their reliability indices are used in the Levenberg-Marquardt (LM) algorithm. In the following, without loss of generality, we describe the method for the case of the thorax segment $R_{SF_{\text{Thorax}}}^{FF}$, where exactly 3 axis estimates are available from the protocol and will provide a means to generalize the method to the other body segments. As a first step, we construct the following vectors:

(3) $$\begin{array}{ll} {\vec{v}}_{\text{ref}}^{FF}={\left[\begin{array}{c} {\vec{\text{x}}}^{FF}\\ {\vec{\text{y}}}^{FF}\\ {\vec{\text{z}}}^{FF} \end{array}\right]}_{9\times 1}, & {\vec{v}}_{\text{est}}^{FF}={\left[\begin{array}{c} {\vec{\text{x}}}^{SF}\\ {\vec{\text{y}}}^{SF}\\ {\vec{\text{z}}}^{SF} \end{array}\right]}_{9\times 1} \end{array}$$

where ${\vec{\text{v}}}_{\text{ref}}^{FF}$ is the set of canonical versors for the FF, *i.e.*, **x⃗***^FF^* = [1 0 0]*^T^*, **y⃗***^FF^* = [0 1 0]*^T^,* **z⃗***^FF^* = [0 0 1]*^T^*, and ${\vec{v}}_{\text{est}}^{SF}$ contains their corresponding estimates expressed in the SF. As shown in Figure 2, each versor in FF is ideally associated with a functional axis, e.g., **y⃗***^FF^* represents the thorax flexion-extension movement. The vectors ${\vec{v}}_{\text{ref}}^{FF}$ and ${\vec{v}}_{\text{est}}^{SF}$ are related by the matrix:

(4) $$Q=\left[\begin{array}{lll} {\hat{R}}_{SF_{\text{Thorax}}}^{FF} & & \\ & {\hat{R}}_{SF_{\text{Thorax}}}^{FF} & \\ & & {\hat{R}}_{SF_{\text{Thorax}}}^{FF} \end{array}\right]\in {\mathbb{R}}^{9\times 9}$$

which is a block diagonal matrix having the rotation matrix estimate repeated on the main diagonal. In the ideal case, when the estimates in ${\vec{v}}_{\text{est}}^{SF}$ are orthogonal and right-handed and **Q** contains the true $R_{SF}^{FF}$, then the equality ${\vec{v}}_{\text{ref}}^{FF}=Q{\vec{v}}_{\text{est}}^{FF}$ is verified. In the real case, the versors composing *^SF^***v⃗***_est_* will most likely be not aligned, rather than orthogonal. Thus, we can define the following error function:

(5) $$\epsilon =({\vec{v}}_{\text{ref}}-Q(\hat{R}){\vec{v}}_{\text{est}})$$

where the symbol, *ϵ*, is the vector of residuals. In order to properly condition the LM algorithm, we used the following cost function:

(6) $$C(\hat{R})=\epsilon^{T}W\epsilon$$

where we introduced a matrix, **W**, of weights built up from the reliability indices, associated with the *^SF^***v⃗***_est_* elements, and defined as:

(7) $$W=\left[\begin{array}{lll} \rho_{x}I_{3\times 3} & & \\ & \rho_{y}I_{3\times 3} & \\ & & \rho_{z}I_{3\times 3} \end{array}\right]$$

If no weights are used, *i.e.*, **W** = **I**, the rotation matrix computed with the LM algorithm will be optimal in the sense of the least squares, *i.e.*, minimizing the sum of squared residuals coming from Equation (5). tion estimate expressed in the form of a unit norm quaternion, *i.e.*, ${\hat{q}}_{SF_{\text{Thorax}}}^{FF}={\left[\begin{array}{llll} w & x & y & z \end{array}\right]}^{T}$. In order to convert the current estimate back to a rotation matrix, we used the following conversion expression:

(8) $$R(q)=\left[\begin{array}{ccc} w^{2}+x^{2}-y^{2}-z^{2} & 2xy-2zw & 2xz+2yw\\ 2xy+2zw & w^{2}+y^{2}-x^{2}-z^{2} & 2yz-2xw\\ 2xz-2yw & 2yz+2xw & w^{2}+z^{2}-x^{2}-y^{2} \end{array}\right]$$

which avoids singularity issues when computing the Jacobian of the cost function. The complete formulation of the LM regression algorithm is given by:

(9) $$\begin{array}{ll} {\hat{q}}_{k+1}^{*} & ={\hat{q}}_{k}-\Delta_{k}\\ {\hat{q}}_{k+1} & =\left\|{\hat{q}}_{k+1}^{*}\right\| \end{array}$$

where ${\hat{q}}_{k+1}^{*}$ is computed from the previous quaternion (**q̂***_k_*) estimate and has to be normalized to enforce the unit-norm condition, which guarantees the estimate to be a rotation in the special orthogonal group, **
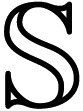

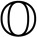
**(3). The variable, Δ*_k_*, represents the regression step at the *k*-iteration, and it is defined as:

(10) $$\begin{array}{c} \Delta_{k}={\left(J^{T}WJ+\lambda \text{diag}(J^{T}WJ)\right)}^{-1}J^{T}W\epsilon \\ J=\partial \epsilon /\partial q \end{array}$$

where **J** is the Jacobian of the error function and λ is a damping parameter, which modulates the algorithm's behavior. Note that the LM is formulated using the more efficient expression by Marquardt for the Fisher matrix (**F** = **J^T^** **W J** + λ *diag*(**J^T^** **W J**)), which reduces the convergence time of the algorithm. We also selected, as a convergence criterion, the percentage variation of the cost function to be under a certain threshold (*ε*):

(11) $$\left|\frac{C{\left(\hat{R}\right)}_{k}-C{\left(\hat{R}\right)}_{k+1}}{C{\left(\hat{R}\right)}_{k}}\right|<\varepsilon$$

Beyond the case of the thorax, the method can be scaled according to the number of vector estimates available, with the constraint of a minimum of 2 estimates in order to unambiguously identify a rotation matrix. In the general case of a number, N, of estimates, the presented matrices and vectors will have the dimension:

(12) v→FFref∈ℝ3N×1,v→SFest∈ℝ3N×1,Q∈ℝ3N×3N,W∈ℝ3N×3N

Finally, further details on the LM algorithm and its implementations can be found in [23].

## 3. Results

Experimental data were collected on the group of 40 children with the methodology described above. The recorded calibration movements were processed in order to extract reference axes together with their reliability measure (*ρ*) of the estimate. We report in Table 1 the values of our reliability index, where the reliability parameter is expressed as the mean and standard deviation on the whole group.

The set of axes estimates and *ρ* values are eventually passed as input to the LM algorithm, where they are included in the matrix of weights, defined in Equation (7). As a pre-condition to the regression, we considered the initial estimate to be the identity rotation, *i.e.*, *q̂* = [1, 0, 0, 0], and we assigned λ = 0.001, which is a typical assumption for this parameter (refer to Appendix A6 in [23]). Moreover, we set the convergence criterion threshold to *ε* = 10^−4^. Given those initialization values, the number of iterations the algorithm undergoes in the average case before attaining convergence is the following, again expressed as the mean and standard deviation:

- Thorax FF: 6.34 ± 0.561 iterations
- Upper arm FF (left and right): 8.36 ± 0.767 iterations
- Forearm FF (left and right): 7.30 ± 0.863 iterations

**Figure 4.**
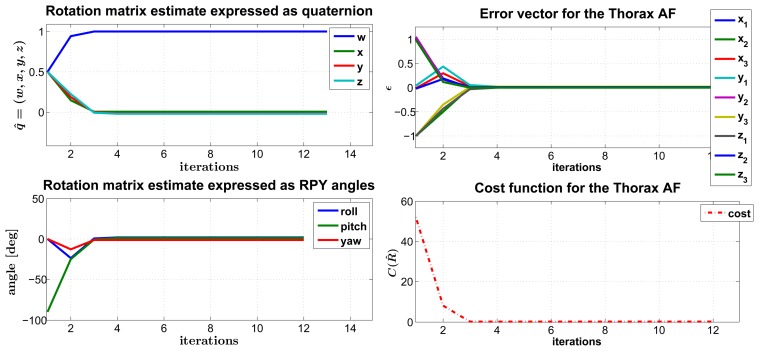
The figure reports the typical converging behavior of the Levenberg-Marquardt (LM) algorithm. On the left, the trend of the $R_{SF}^{FF}$ matrix estimate relative to the FFThorax is shown, respectively expressed in the form of a unit-norm quaternion (top) and as Euler roll, pitch and yaw angles (bottom). On the right, the plots represents the error vector (top) and the cost function (bottom), respectively defined in Equations (5) and (6).

As an example of the converging behavior of the LM algorithm, we reported the case of the thorax FF rotation matrix in Figure 4. Besides, in this particular case, we stressed that the algorithm performance gives random initialization values as input, and we still observed the attainment of convergence after a moderate number of iterations (12 in the example).

**Figure 5.**
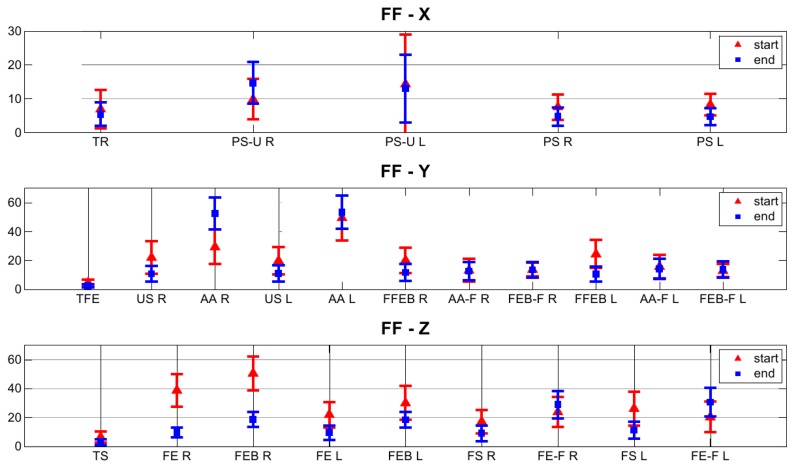
Comparison of the differences between pairs (**v⃗***_ref_*, **v⃗***_est_*) of axes estimates and their FF matches, computed at the beginning and at the end of the LM regression (refer to Section 2.2.1. for the meaning of the acronyms). As a distance metric, we considered the angle between each pair of vectors, computed as the arccosine of their dot product.

With the purpose of gaining further insight into the behavior of our regression algorithm, we focused on the error function, particularly on the vector of residuals, *ϵ*. As formalized in Equation (5), this vector is made of the Euclidean differences between each FF canonical axis (*i.e.*, the versors **x̂**, **ŷ**, and **ẑ**) and its corresponding estimate, both expressed in the FF system of coordinates. The aim of the regression algorithm would be that of mitigating these differences, by making matched pairs of vectors (**v⃗***_ref_*, **v⃗***_est_*) pointing to approximately the same directions in ℝ^3^ space, *i.e.*, as close as possible, in accordance with the mathematical constraints associated with the rotation matrix, ${\hat{R}}_{SF}^{FF}$ (e.g., the orthogonality of the column vectors). In our weighted formulation of the LM algorithm, some pairs of vectors are expected to get closer than others, depending on the value of their reliability index. In addition, the pairs that get closer are also the ones that mostly affect the computation of the transformation matrix ${\hat{R}}_{SF}^{FF}$. In order to visualize this effect, we made a comparison of the values of these differences at the first step, when ${\hat{R}}_{SF}^{FF}=I_{3\times 3}$, and at the convergence of the regression algorithm, ${\hat{R}}_{SF}^{FF}=\underset{R}{\arg\min}(C(R))$. As a distance metric, we considered:

(13) $$d({\vec{v}}_{\text{ref}},{\vec{v}}_{\text{est}})=\text{acos}({\vec{v}}_{\text{ref}}^{T}{\vec{v}}_{\text{est}})$$

which is the angle between each pair of vectors. The analysis was extended to the whole set of 40 children, and we reported the results, expressed as the mean and standard deviation, for each movement, in Figure 5.

## 4. Discussion and Conclusions

This paper describes a novel calibration protocol for the kinematic tracking of the thorax and upper limbs with M-IMU wearable sensors, designed to be used with children. This method allows a user to define functional coordinate systems (FF) that are fixed on the body segments and to estimate the relation between an M-IMU sensor's frame and its corresponding body segment's FF. The proposed calibration procedure itself is made of a list of movements and a methodology to elaborate sensor data, in order to compute a rotation matrix relating each SF to the corresponding, arbitrarily-defined FF (*i.e.*, $R_{SF}^{FF}$). Selected movements have the two-fold purpose of identifying a sufficient number of non-aligned axes on each defined FF, at least two, and conforming to the constraints of being easy to perform and short in duration, in order to avoid the fatigue of the children and to reduce the overall duration of experimental sessions.

In fact, as highlighted in our previous study [20] and as we also observed during the experimentation, some movements are easier to perform for children (6–7 yo) than others: as an example, the thorax lateral flexion, used in similar calibration protocols with adults [18], resulted in an improper choice for children. Furthermore, given the difficulty to maintain children's attention for a long time compared to adults, we tried to optimize the calibration protocol in order to limit the duration while maintaining a substantial number of functional axis estimates, as suggested in [15]. In addition, the protocol is presented as a game of imitation: an adult plays the role of the coach, and the subject is asked to mirror his movements. This experimental methodology is specific for usage with children, as it brings the two-fold benefit of: (i) making it easier for children to understand how the movements should be performed; and (ii) having an adult checking the correctness of the movement. No difficulties were encountered in the experimentation with the proposed list, and in all but the thorax case, a redundant number of functional axes were identified, referring to the minimum of a pair of non-aligned axes that is necessary in order to estimate a rotation matrix. Moreover, our novel data analysis approach eliminates the typical need of a segmentation process, which usually involves a rest period between any two phases of a rotation movement, e.g., to differentiate the flexion from the extension phase, as reported in [15,16,18]. Thus, with our methodology, the duration of this part of the protocol is further reduced (*i.e.*, lasting 15 min).

In view of the necessity that may arise of pruning the list of movements to the minimum of two axes per FF, we introduced the reliability index, *ρ*, which is defined in Equation (2), and we used it to qualify the estimated axes, as reported in Table 1. The index of reliability is computed from measurement matrices containing repetitions (from nine to 12) of the same functional movement/reference posture and, thus, gives an indication of the precision of the child's performance. In the ideal case of a noiseless sensing unit, the maximum value of the index (*i.e.,* one) is obtained when exactly the same axis of rotation is involved in each repetition of the movement. Instead, the minimum value of the index (*i.e.,* 1/3) is mathematically obtained in the limit case when each repetition of the movement belongs to a different axis of rotation and those axes are orthogonal, e.g., that would be the case in which a subject is asked to perform a flexion-extension of the upper arm three times and he instead performs a flexion-extension first, then an ab- and ad-duction and, finally, a pronosupination. Further, high precision in the execution of a movement translates into the high repeatability of the estimated reference axis.

The proposed list of movements deliberately included the same physiological movement (*i.e.,* flexion and extension) executed with or without the support of a rigid bar. The reason for that is the possibility to evaluate if, as expected from intuition, the introduction of an external support to further facilitate children's coordination improves the reliability of some movements. From the reported table, the reliability index indicates that the supported movement is better than the other. In addition, we carried out statistical analysis using a paired *t*-test on the normally distributed reliability datasets, and we obtained a significant difference (*p* < 0.05) both for the forearm FFs and for the upper arm FFs.

In Figure 5, we proposed a visualization of the residual distance between vector pairs (**v⃗***_ref_*, **v⃗***_est_*), as defined in Equation (13), at the beginning and at the end of the LM regression and for each movement in the calibration protocol. We observed a general trend of the pairs of axes estimates and corresponding FF reference axes, to reduce their angular distance at the end of the regression. If more than one estimate for the same FF axis is available, the regression algorithm will favor the one with the higher reliability value. For instance, this is true for the case of the supine posture (*ρ* = 0.98 and *ρ* = 0.99) *versus* the abduction and adduction movement (*ρ* = 0.58 and *ρ* = 0.59) of the upper arms on the t-axis estimation of the associated FF. The thorax was the body segment with the overall highest reliability and with the lowest residual distance between the pairs (**v⃗***_ref_*, **v⃗***_est_*) in the rotation matrix estimation, respectively, 5.39°, 2.56° and 2.86° for the x, y and z component. This is due to a proper choice of the calibration movements for the thorax, with reference to both the repeatability and the fact that the set of axes estimated during each movement/reference pose is close to an orthogonal frame. Moreover, this result is in accordance with what was discussed in [18], where the functional frame built on the thorax is even proven to be the most compatible with the anatomical frame defined by the ISBrecommendations [24]. The pronosupination of the forearm is the most reliable movement for the functional part of this specific FF, and its associated axis is the most repeatable, in agreement with similar studies in the literature [18,20].

Furthermore, the residual angular distance for the pairs (**v⃗***_ref_*, **v⃗***_est_*) amounts to 4.68° for the left and 4.72° for the right forearm.

The standard way to estimate the rotation matrix, $R_{SF}^{FF}$, in the literature [15,16,18,24] is that of using a single pair of non-aligned axis estimates to get an orthonormal frame via successive vector products. This method is referred to in the literature as the TRIAD (Tri-axial Attitude Determination) algorithm and was originally proposed as a solution to Wahba's problem [25]. Our approach to $R_{SF}^{FF}$ estimation overcomes the known limitations of the TRIAD algorithm, in the sense that it is capable of accommodating more than two axis estimates, is not sensible to the order at which the axis estimates are considered and, more importantly, can exploit all the available information (*i.e.*, both the axis direction and its repeatability measure).

**Figure 6.**
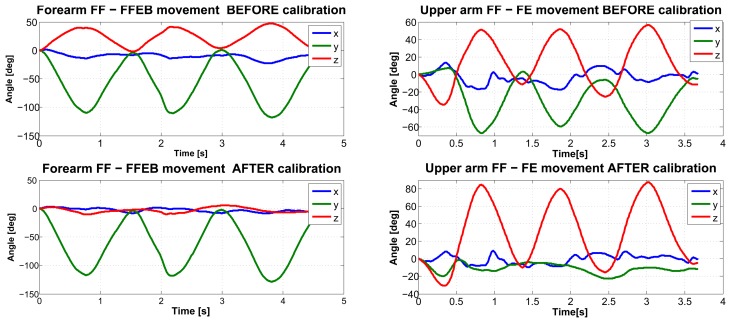
An example of kinematic reconstruction of the right arm during the flexion-extension (FE) movement and the supported forearm flexion-extension (FFEB) with the proposed calibration procedure.

Finally, as an outcome to the method we reported in Figure 6, we present an example of the kinematic reconstruction for the right upper limb. Though evidence exists of a substantial reduction in kinematic cross-talking for single joint movements with the proposed calibration methodology, we believe that future research endeavors should focus on the comparison with data collected using optical motion capture systems, which are considered as the gold standard.

## Figures and Tables

**Table 1.** The table reports the values for the reliability index, *ρ*, which is a dimensionless number in the range, [ $\frac{1}{3}$, 1], relative to each calibration movement. The value is expressed as a mean (±1 SD) computed on our group of 40 children.

**Calibration Movement**	**Thorax**	**Upper Arm L**	**Upper Arm R**	**Forearm L**	**Forearm R**

Supine on the ground	0.98 ±0.006	0.98 ±0.018	0.99 ±0.013	0.97 ±0.022	0.98 ±0.018
Thorax rotation on the transverse plane	0.79 ±0.043	⫽	⫽	⫽	⫽
Thorax flexion-extension	0.78 ±0.050	⫽	⫽	⫽	⫽
Forearm flexion-extension with bar	⫽	⫽	⫽	0.74 ±0.044	0.74 ±0.046
Upper arm flexion-extension with bar	⫽	0.63 ±0.044	0.63 ±0.041	0.68 ±0.048	0.69 ±0.049
Upper arm flexion-extension	⫽	0.60 ±0.042	0.61 ±0.038	0.55 ±0.031	0.57 ±0.035
Upper arm abb-adduction	⫽	0.58 ±0.035	0.59 ±0.028	0.53 ±0.034	0.55 ±0.031
Forearm prono-supination	⫽	0.67 ±0.089	0.66 ±0.085	0.83 ±0.053	0.84 ±0.050
